# Supplementary material for: Effect of preterm birth on early neonatal, late neonatal, and postneonatal mortality in India
Source: PLOS Glob Public Health. 2022 Jun 28;2(6):e0000205. doi: 10.1371/journal.pgph.0000205 (PMC10021707; doi:10.1371/journal.pgph.0000205)
Supplement: S1 Fig — Note: 1. Odds ratios are significant at p < 0.05. 2. Odds ratios are adjusted for birth order, index child c-section, index birth wanted, sex of child, mother’s age at conception, mother’s height, mother’s schooling, caste, religion, wealth quintiles, urban-rural residence, and state-region. Neonatal deaths (NND), infant deaths (IND). (DOC) [file pgph.0000205.s001.doc]

**S1 Fig. Adjusted odds ratio of NND and IND for preterm birth, NFHS-4, India, 2015-16.**


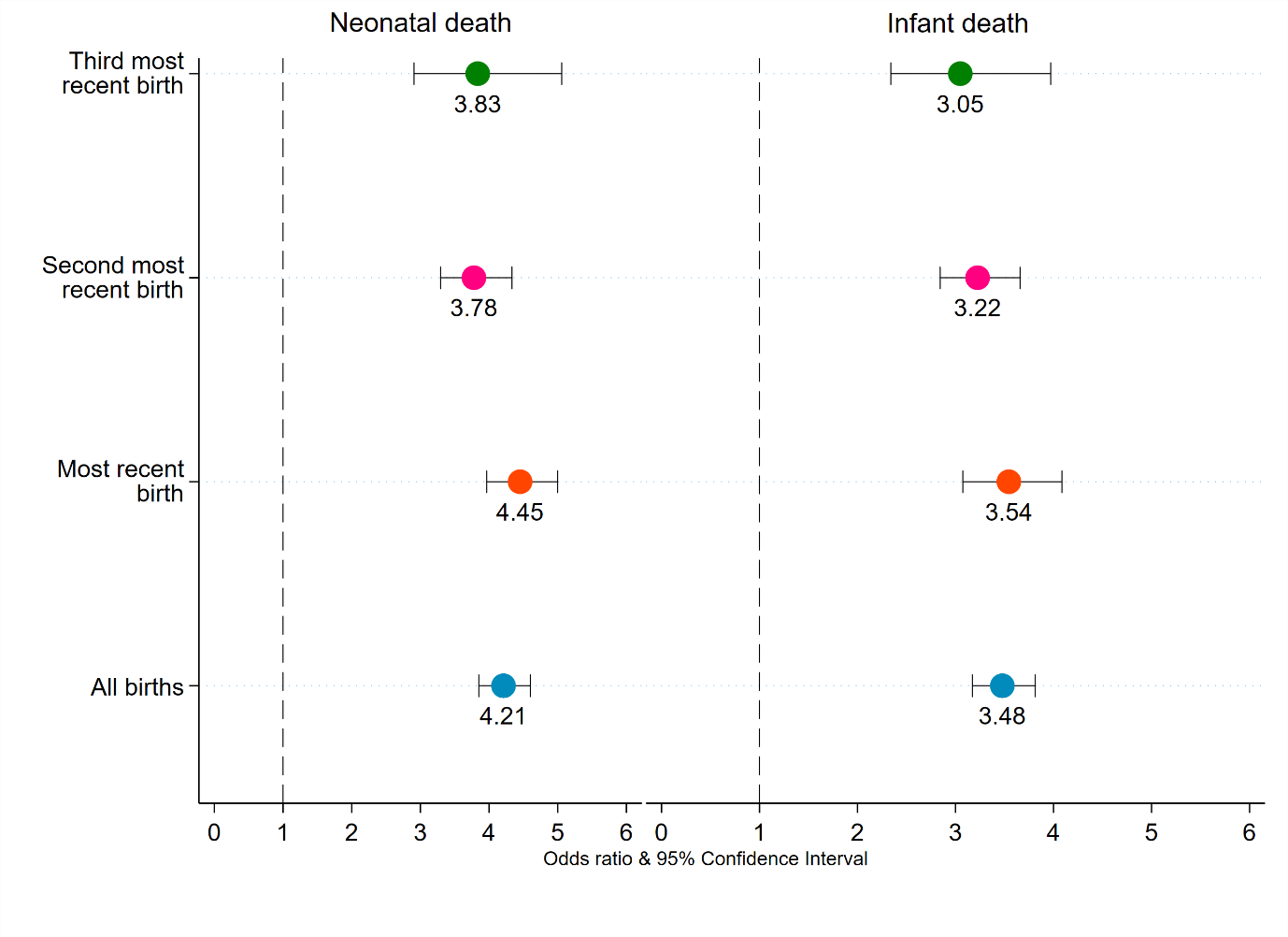


***Note:*** *1. Odds ratios are significant at p < 0.05. 2. Odds ratios are adjusted for birth order, index child c-section, index birth wanted, sex of the child, mother's age at conception, mother's height, mother's schooling, caste, religion, wealth quintiles, urban-rural residence, and state-region. Neonatal deaths (NND), infant deaths (IND)*
