# Supplementary material for: Effect of preterm birth on early neonatal, late neonatal, and postneonatal mortality in India
Source: PLOS Glob Public Health. 2022 Jun 28;2(6):e0000205. doi: 10.1371/journal.pgph.0000205 (PMC10021707; doi:10.1371/journal.pgph.0000205)
Supplement: S1 Table — (DOC) [file pgph.0000205.s002.doc]

| **S1 Table. States included in various categories of state-region.** | |
| --- | --- |
| **Category** | **States included** |
| North | Chandigarh, Haryana, Himachal Pradesh, Jammu and Kashmir, Delhi, Rajasthan, Punjab, Uttarakhand |
| Central | Chhattisgarh, Madhya Pradesh, Uttar Pradesh |
| East | Bihar, Jharkhand, Odisha, West Bengal |
| Northeast | Arunachal Pradesh, Assam, Manipur, Meghalaya, Mizoram, Nagaland, Sikkim, Tripura |
| West | Dadra and Nagar Haveli, Daman and Diu, Goa, Gujarat, Maharashtra |
| South | Andaman and Nicobar, Andhra Pradesh, Karnataka, Kerala, Lakshadweep, Puducherry, Tamil Nadu, Telangana |
