# Supplementary material for: Effect of preterm birth on early neonatal, late neonatal, and postneonatal mortality in India
Source: PLOS Glob Public Health. 2022 Jun 28;2(6):e0000205. doi: 10.1371/journal.pgph.0000205 (PMC10021707; doi:10.1371/journal.pgph.0000205)
Supplement: S2 Table — Notes: 1. Fourth most recent births in the past five years were excluded due to very small sample sizes. 2. 95% confidence intervals are shown in the parenthesis. 3. Early neonatal death (ENND), late neonatal death (LNND), and postneonatal death (PNND). (DOC) [file pgph.0000205.s003.doc]

| **S2 Table. Percentage of preterm birth and ENND, percentage of preterm birth and LNND, and percentage of preterm birth and PNND by sequence of birth among births in the past five years, NFHS-4, India, 2015-16.** | | | | | | | | |
| --- | --- | --- | --- | --- | --- | --- | --- | --- |
| **Sequence of births** | **ENND sample** | |  | **LNND sample** | |  | **PNND sample** | |
|  | **Preterm birth** | **ENND** |  | **Preterm birth** | **LNND** |  | **Preterm birth** | **PNND** |
| All births | 6.8% (6.5,7.0) | 2.4% (2.3,2.5) |  | 6.8% (6.5,7.0) | 0.4% (0.4,0.5) |  | 6.6% (6.4,6.9) | 1.0%  (0.9,1.1) |
| Most recent births | 6.5% (6.2,6.7) | 1.5% (1.4,1.5) |  | 6.5% (6.2,6.7) | 0.2% (0.2,0.3) |  | 6.2% (5.9,6.4) | 0.6%  (0.6,0.7) |
| Second most recent birth | 7.3% (6.9,7.6) | 4.2% (4.0,4.4) |  | 7.3% (6.9,7.6) | 0.8% (0.7,0.9) |  | 7.2% (6.9,7.6) | 1.7%  (1.5,1.8) |
| Third most recent birth | 9.4% (8.5,10.4) | 9.2% (8.3,10.3) |  | 9.4% (8.5,10.4) | 1.6% (1.3,2.0) |  | 9.4% (8.5,10.4) | 3.1%  (2.7,3.7) |
| *Notes: 1. Fourth most recent births in the past five years were excluded due to very small sample sizes.*  *2. 95% confidence intervals are shown in the parenthesis.*  *3.* Note. *Early neonatal death (ENND), late neonatal death (LNND), and postneonatal death (PNND)* | | | | | | | | |
