# Supplementary material for: Effect of preterm birth on early neonatal, late neonatal, and postneonatal mortality in India
Source: PLOS Glob Public Health. 2022 Jun 28;2(6):e0000205. doi: 10.1371/journal.pgph.0000205 (PMC10021707; doi:10.1371/journal.pgph.0000205)
Supplement: S3 Table — Note: 1. Fourth most recent births in the past five years were excluded due to very small sample sizes. 2. Early neonatal death (ENND), late neonatal death (LNND), and postneonatal death (PNND). (DOC) [file pgph.0000205.s004.doc]

| **S3 Table. ENND, LNND, and PNND by preterm birth according to sequence of birth among births in the past five years, NFHS-4, India, 2015-16.** | | | | | | | |
| --- | --- | --- | --- | --- | --- | --- | --- |
| **Sequence of births** | **Preterm birth** | **ENND** | **ENND (%)** | **LNND** | **LNND (%)** | **PNND** | **PNND (%)** |
| **All births** | | | | | | | |
|  | No | 4489 | 1.98% | 804 | 0.4% | 2010 | 1.0% |
|  | Yes | 1349 | 8.22% | 203 | 1.4% | 265 | 1.8% |
| **Most recent birth** | | | | | | | |
|  | No | 2041 | 1.19% | 340 | 0.2% | 768 | 0.6% |
|  | Yes | 625 | 5.41% | 85 | 0.9% | 102 | 1.2% |
| **Second most recent birth** | | | | | | | |
|  | No | 1882 | 3.57% | 350 | 0.7% | 975 | 1.6% |
|  | Yes | 545 | 12.65% | 84 | 2.4% | 130 | 2.6% |
| **Third most recent birth** | | | | | | | |
|  | No | 510 | 7.59% | 104 | 1.4% | 244 | 3.2% |
|  | Yes | 160 | 24.93% | 30 | 4.0% | 25 | 2.5% |
| ***Note****:* ***1.*** *Fourth most recent births in the past five years were excluded due to very small sample sizes.*  *2. Early neonatal death (ENND), late neonatal death (LNND), and postneonatal death (PNND)* | | | | | | | |
