# Supplementary material for: Effect of preterm birth on early neonatal, late neonatal, and postneonatal mortality in India
Source: PLOS Glob Public Health. 2022 Jun 28;2(6):e0000205. doi: 10.1371/journal.pgph.0000205 (PMC10021707; doi:10.1371/journal.pgph.0000205)
Supplement: S4 Table — Note: OR: odds ratio; * p < 0.05; CI: Confidence interval. (DOC) [file pgph.0000205.s005.doc]

| **S4 Table. Adjusted odds ratio of early neonatal deaths (ENND) for preterm birth, NFHS-4, India, 2015-16.** | | | | |
| --- | --- | --- | --- | --- |
| **Variable & category** | **All births** | **Most recent birth** | **Second most recent birth** | **Third most recent birth** |
|  | **OR (95%CI)** | **OR (95%CI)** | **OR (95%CI)** | **OR (95%CI)** |
| **Index birth preterm** |  |  |  |  |
| No (reference) | 1.00 | 1.00 | 1.00 | 1.00 |
| Yes | 4.15*(3.79,4.56) | 4.43*(3.92,5.01) | 3.68*(3.20,4.23) | 3.73*(2.77,5.02) |
| **Birth order (BO) and birth interval (BI)** |  |  |  |  |
| First birth order | 1.95*(1.67,2.26) | 1.64*(1.35,2.00) | 1.45*(1.10,1.90) | 1.07(0.58,1.99) |
| BO 2 or 3 and BI <24 months | 1.75*(1.5,2.04) | 1.38*(1.12,1.69) | 1.92*(1.46,2.51) | 1.61(0.87,2.99) |
| BO 2 or 3 and BI 2 or 3 and ≥24 months | 0.94(0.82,1.08) | 0.89(0.75,1.06) | 1.23(0.95,1.59) | 0.63(0.33,1.18) |
| BO ≥4 and BI <24 months | 2.19*(1.87,2.57) | 1.90*(1.55,2.34) | 2.20*(1.66,2.93) | 1.57(0.76,3.23) |
| BO ≥4 and BI ≥24 months (reference) | 1.00 | 1.00 | 1.00 | 1.00 |
| **Index child c-section** |  |  |  |  |
| No (reference) | 1.00 | 1.00 | 1.00 | 1.00 |
| Yes | 0.93(0.82,1.05) | 1.10(0.94,1.30) | 1.11(0.89,1.38) | 1.27(0.83,1.95) |
| **Index birth wanted** |  |  |  |  |
| No | 1.02(0.91,1.15) | 1.15(0.98,1.34) | 0.98(0.80,1.19) | 1.38(0.91,2.09) |
| Yes (reference) | 1.00 | 1.00 | 1.00 | 1.00 |
| **Sex of child** |  |  |  |  |
| Male (reference) | 1.00 | 1.00 | 1.00 | 1.00 |
| Female | 0.75*(0.70,0.80) | 0.86*(0.78,0.95) | 0.61*(0.54,0.68) | 0.38*(0.30,0.47) |
| **Mother's age at conception** |  |  |  |  |
| <20 years | 1.22*(1.10,1.36) | 1.03(0.87,1.22) | 1.16*(1.01,1.34) | 1.39*(1.05,1.83) |
| 20-24 years (reference) | 1.00 | 1.00 | 1.00 | 1.00 |
| 25-29 years | 0.94(0.85,1.04) | 1.02(0.89,1.16) | 1.07(0.92,1.26) | 1.11(0.80,1.55) |
| ≥30 years | 1.22*(1.07,1.39) | 1.49*(1.27,1.75) | 1.33*(1.06,1.69) | 0.96(0.54,1.70) |
| **Mother's height** |  |  |  |  |
| <145cm | 1.39*(1.26,1.53) | 1.33*(1.17,1.51) | 1.34*(1.16,1.54) | 1.44*(1.09,1.89) |
| ≥145cm (reference) | 1.00 | 1.00 | 1.00 | 1.00 |
| Refused/Others/Missing | 1.02(0.74,1.40) | 0.81(0.53,1.26) | 1.27(0.74,2.16) | 1.70(0.64,4.49) |
| **Mother's schooling** |  |  |  |  |
| No schooling | 1.19*(1.08,1.32) | 1.30*(1.14,1.50) | 0.91(0.79,1.06) | 0.56*(0.42,0.74) |
| Primary | 1.28*(1.15,1.43) | 1.41*(1.21,1.64) | 1.04(0.88,1.23) | 0.80(0.59,1.08) |
| Secondary or Higher (reference) | 1.00 | 1.00 | 1.00 | 1.00 |
| **Caste** |  |  |  |  |
| Scheduled Caste | 1.11(0.98,1.26) | 1.17(0.99,1.39) | 0.99(0.82,1.21) | 0.97(0.66,1.43) |
| Scheduled Tribe | 0.95(0.83,1.10) | 0.99(0.82,1.20) | 0.98(0.78,1.23) | 0.86(0.52,1.44) |
| Other Backward Class | 1.07(0.96,1.19) | 1.13(0.97,1.31) | 0.97(0.82,1.15) | 0.96(0.67,1.37) |
| Others (reference) | 1.00 | 1.00 | 1.00 | 1.00 |
| **Religion** |  |  |  |  |
| Hindu | 1.22*(1.01,1.46) | 1.16(0.90,1.50) | 1.40*(1.01,1.93) | 1.03(0.60,1.76) |
| Muslim | 1.18(0.95,1.45) | 1.16(0.87,1.54) | 1.21(0.85,1.74) | 0.66(0.36,1.23) |
| Others (reference) | 1.00 | 1.00 | 1.00 | 1.00 |
| **Wealth quintiles** |  |  |  |  |
| Poorest | 1.91*(1.58,2.31) | 2.15*(1.66,2.79) | 1.33*(1.01,1.77) | 1.12(0.62,2.02) |
| Poorer | 1.77*(1.47,2.12) | 2.00*(1.57,2.55) | 1.25(0.96,1.63) | 1.29(0.73,2.27) |
| Middle | 1.69*(1.42,2.01) | 1.79*(1.41,2.27) | 1.38*(1.06,1.80) | 1.13(0.66,1.94) |
| Richer | 1.35*(1.13,1.61) | 1.26(0.98,1.61) | 1.20(0.92,1.55) | 1.26(0.70,2.28) |
| Richest (reference) | 1.00 | 1.00 | 1.00 | 1.00 |
| **Urban-rural residence** |  |  |  |  |
| Urban (reference) | 1.00 | 1.00 | 1.00 | 1.00 |
| Rural | 1.20*(1.07,1.35) | 1.18*(1.02,1.38) | 1.28*(1.08,1.51) | 1.12(0.79,1.59) |
| **State-region** |  |  |  |  |
| North | 1.54*(1.31,1.81) | 1.60*(1.28,2.00) | 1.51*(1.17,1.96) | 1.28(0.81,2.02) |
| Centre | 1.91*(1.65,2.21) | 1.81*(1.47,2.23) | 2.00*(1.59,2.52) | 1.34(0.88,2.05) |
| East | 1.38*(1.18,1.61) | 1.29*(1.04,1.61) | 1.82*(1.42,2.32) | 1.21(0.77,1.90) |
| Northeast | 1.36*(1.13,1.63) | 1.17(0.90,1.52) | 2.15*(1.60,2.87) | 1.29(0.70,2.39) |
| West | 1.18(0.95,1.47) | 1.08(0.81,1.44) | 1.12(0.80,1.57) | 1.85(0.96,3.58) |
| South (reference) | 1.00 | 1.00 | 1.00 | 1.00 |
| ***Note****: OR: odds ratio; * p < 0.05; CI: Confidence interval* | | | | |
