# Supplementary material for: Effect of preterm birth on early neonatal, late neonatal, and postneonatal mortality in India
Source: PLOS Glob Public Health. 2022 Jun 28;2(6):e0000205. doi: 10.1371/journal.pgph.0000205 (PMC10021707; doi:10.1371/journal.pgph.0000205)
Supplement: S5 Table — Note: OR: odds ratio; * p < 0.05; CI: Confidence interval. (DOC) [file pgph.0000205.s006.doc]

| **S5 Table. Adjusted odds ratio of late neonatal deaths (LNND) for preterm birth, NFHS-4, India, 2015-16.** | | | | |
| --- | --- | --- | --- | --- |
| **Variable & category** | **All births** | **Most recent birth** | **Second most recent birth** | **Third most recent birth** |
|  | **OR (95%CI)** | **OR (95%CI)** | **OR (95%CI)** | **OR (95%CI)** |
| **Index birth preterm** |  |  |  |  |
| No (reference) | 1.00 | 1.00 | 1.00 | 1.00 |
| Yes | 3.77*(2.98,4.77) | 3.97*(2.90,5.45) | 3.39*(2.29,5.03) | 2.78*(1.69,4.59) |
| **Birth order (BO) and birth interval (BI)** |  |  |  |  |
| First birth order | 2.00*(1.38,2.88) | 2.14*(1.34,3.42) | 0.94(0.50,1.74) | 1.21(0.30,4.96) |
| BO 2 or 3 and BI <24 months | 2.51*(1.74,3.61) | 1.70(0.97,2.98) | 2.24*(1.21,4.15) | 2.93(0.75,11.5) |
| BO 2 or 3 and BI 2 or 3 and ≥24 months | 1.38(0.97,1.96) | 1.41(0.95,2.10) | 1.51(0.78,2.92) | 0.77(0.19,3.14) |
| BO ≥4 and BI <24 months | 3.11*(2.17,4.47) | 3.16*(1.99,5.04) | 2.00*(1.04,3.86) | 3.41(0.95,12.27) |
| BO ≥4 and BI ≥24 months (reference) | 1.00 | 1.00 | 1.00 | 1.00 |
| **Index child c-section** |  |  |  |  |
| No (reference) | 1.00 | 1.00 | 1.00 | 1.00 |
| Yes | 0.87(0.62,1.22) | 1.28(0.91,1.80) | 1.01(0.53,1.93) | 0.24*(0.07,0.87) |
| **Index birth wanted** |  |  |  |  |
| No | 1.04(0.80,1.36) | 1.25(0.89,1.74) | 0.99(0.60,1.61) | 1.42(0.69,2.92) |
| Yes (reference) | 1.00 | 1.00 | 1.00 | 1.00 |
| **Sex of child** |  |  |  |  |
| Male (reference) | 1.00 | 1.00 | 1.00 | 1.00 |
| Female | 0.88(0.74,1.05) | 1.08(0.85,1.36) | 0.60*(0.45,0.80) | 0.60*(0.40,0.92) |
| **Mother's age at conception** |  |  |  |  |
| <20 years | 1.40*(1.10,1.78) | 1.63*(1.12,2.36) | 1.18(0.82,1.70) | 1.13(0.66,1.95) |
| 20-24 years (reference) | 1.00 | 1.00 | 1.00 | 1.00 |
| 25-29 years | 1.14(0.90,1.46) | 1.57*(1.14,2.18) | 1.17(0.80,1.72) | 1.03(0.55,1.93) |
| ≥30 years | 1.53*(1.09,2.13) | 1.97*(1.36,2.85) | 2.08*(1.06,4.10) | 1.20(0.45,3.20) |
| **Mother's height** |  |  |  |  |
| <145cm | 1.64*(1.24,2.17) | 1.68*(1.22,2.32) | 1.58(0.96,2.61) | 1.16(0.66,2.04) |
| ≥145cm (reference) | 1.00 | 1.00 | 1.00 | 1.00 |
| Refused/Others/Missing | 0.98(0.46,2.09) | 1.04(0.44,2.44) | 0.79(0.18,3.53) | 2.24(0.33,15.01) |
| **Mother's schooling** |  |  |  |  |
| No schooling | 1.14(0.91,1.43) | 1.28(0.91,1.79) | 0.80(0.55,1.16) | 0.59(0.34,1.02) |
| Primary | 1.15(0.87,1.53) | 1.15(0.79,1.67) | 0.97(0.60,1.58) | 0.70(0.38,1.31) |
| Secondary or Higher (reference) | 1.00 | 1.00 | 1.00 | 1.00 |
| **Caste** |  |  |  |  |
| Scheduled Caste | 1.18(0.86,1.62) | 1.32(0.91,1.93) | 1.10(0.63,1.90) | 0.81(0.42,1.58) |
| Scheduled Tribe | 1.01(0.73,1.40) | 1.12(0.72,1.75) | 1.03(0.60,1.75) | 0.78(0.33,1.82) |
| Other Backward Class | 1.11(0.85,1.46) | 1.25(0.90,1.74) | 0.99(0.62,1.61) | 0.93(0.50,1.73) |
| Others (reference) | 1.00 | 1.00 | 1.00 | 1.00 |
| **Religion** |  |  |  |  |
| Hindu | 1.18(0.62,2.23) | 1.15(0.63,2.11) | 1.30(0.35,4.84) | 0.77(0.27,2.24) |
| Muslim | 0.94(0.49,1.83) | 1.11(0.57,2.18) | 0.64(0.17,2.43) | 0.68(0.20,2.34) |
| Others (reference) | 1.00 | 1.00 | 1.00 | 1.00 |
| **Wealth quintiles** |  |  |  |  |
| Poorest | 1.58*(1.01,2.50) | 2.13*(1.25,3.62) | 0.96(0.43,2.13) | 0.97(0.38,2.45) |
| Poorer | 1.37(0.88,2.13) | 1.84*(1.11,3.04) | 0.92(0.42,1.99) | 0.79(0.32,1.96) |
| Middle | 1.34(0.84,2.13) | 1.72*(1.05,2.82) | 1.08(0.47,2.47) | 0.49(0.19,1.29) |
| Richer | 1.07(0.65,1.75) | 1.12(0.63,1.97) | 1.04(0.44,2.46) | 0.47(0.19,1.19) |
| Richest (reference) | 1.00 | 1.00 | 1.00 | 1.00 |
| **Urban-rural residence** |  |  |  |  |
| Urban (reference) | 1.00 | 1.00 | 1.00 | 1.00 |
| Rural | 0.88(0.68,1.14) | 0.98(0.70,1.38) | 0.77(0.52,1.15) | 1.15(0.58,2.25) |
| **State-region** |  |  |  |  |
| North | 2.42*(1.55,3.78) | 2.42*(1.33,4.42) | 2.34*(1.08,5.06) | 1.64(0.54,4.99) |
| Centre | 3.15*(2.10,4.74) | 2.93*(1.66,5.17) | 2.85*(1.42,5.70) | 1.82(0.61,5.46) |
| East | 1.69*(1.09,2.61) | 1.31(0.73,2.35) | 2.13(0.99,4.57) | 1.25(0.38,4.11) |
| Northeast | 1.76(0.99,3.14) | 1.59(0.78,3.25) | 2.51(0.81,7.83) | 0.57(0.06,5.54) |
| West | 1.76(0.95,3.26) | 1.10(0.55,2.19) | 2.78*(1.09,7.09) | 0.31(0.06,1.53) |
| South (reference) | 1.00 | 1.00 | 1.00 | 1.00 |
| ***Note****: OR: odds ratio; * p < 0.05; CI: Confidence interval* | | | | |
