# Supplementary material for: Effect of preterm birth on early neonatal, late neonatal, and postneonatal mortality in India
Source: PLOS Glob Public Health. 2022 Jun 28;2(6):e0000205. doi: 10.1371/journal.pgph.0000205 (PMC10021707; doi:10.1371/journal.pgph.0000205)
Supplement: S6 Table — Note: OR: odds ratio; * p < 0.05; CI: Confidence interval. (DOC) [file pgph.0000205.s007.doc]

| **S6 Table. Adjusted odds ratio of postneonatal deaths (PNND) for preterm birth, NFHS-4, India, 2015-16.** | | | | |
| --- | --- | --- | --- | --- |
| **Variable & category** | **All births** | **Most recent birth** | **Second most recent birth** | **Third most recent birth** |
|  | **OR (95%CI)** | **OR (95%CI)** | **OR (95%CI)** | **OR (95%CI)** |
| **Index birth preterm** |  |  |  |  |
| No (reference) | 1.00 | 1.00 | 1.00 | 1.00 |
| Yes | 1.67*(1.40,1.99) | 2.03*(1.51,2.73) | 1.46*(1.16,1.84) | 0.94(0.62,1.44) |
| **Birth order (BO) and birth interval (BI)** |  |  |  |  |
| First birth order | 1.08(0.83,1.40) | 0.65(0.41,1.03) | 1.05(0.72,1.52) | 0.63(0.32,1.25) |
| BO 2 or 3 and BI <24 months | 1.59*(1.23,2.04) | 0.93(0.61,1.41) | 2.05*(1.43,2.96) | 1.46(0.74,2.90) |
| BO 2 or 3 and BI 2 or 3 and ≥24 months | 0.93(0.74,1.16) | 0.65*(0.47,0.90) | 1.46*(1.03,2.06) | 0.73(0.36,1.49) |
| BO ≥4 and BI <24 months | 2.68*(2.14,3.35) | 1.99*(1.45,2.72) | 3.17*(2.21,4.54) | 2.77*(1.38,5.56) |
| BO ≥4 and BI ≥24 months (reference) | 1.00 | 1.00 | 1.00 | 1.00 |
| **Index child c-section** |  |  |  |  |
| No (reference) | 1.00 | 1.00 | 1.00 | 1.00 |
| Yes | 1.04(0.83,1.30) | 1.34(0.96,1.86) | 1.20(0.84,1.72) | 1.14(0.67,1.93) |
| **Index birth wanted** |  |  |  |  |
| No | 1.05(0.86,1.28) | 1.02(0.76,1.36) | 1.26(0.94,1.69) | 0.85(0.48,1.47) |
| Yes (reference) | 1.00 | 1.00 | 1.00 | 1.00 |
| **Sex of child** |  |  |  |  |
| Male (reference) | 1.00 | 1.00 | 1.00 | 1.00 |
| Female | 1.08(0.96,1.21) | 1.33*(1.09,1.62) | 0.85*(0.73,0.99) | 0.54*(0.42,0.69) |
| **Mother's age at conception** |  |  |  |  |
| <20 years | 1.48*(1.22,1.80) | 1.37(0.88,2.14) | 1.46*(1.16,1.84) | 1.21(0.88,1.65) |
| 20-24 years (reference) | 1.00 | 1.00 | 1.00 | 1.00 |
| 25-29 years | 1.11(0.95,1.29) | 1.09(0.83,1.42) | 1.47*(1.21,1.80) | 0.77(0.52,1.12) |
| ≥30 years | 1.17(0.96,1.43) | 1.26(0.93,1.70) | 1.53*(1.12,2.09) | 1.06(0.63,1.79) |
| **Mother's height** |  |  |  |  |
| <145cm | 1.16(0.98,1.36) | 1.23(0.96,1.58) | 1.07(0.84,1.36) | 1.15(0.82,1.62) |
| ≥145cm (reference) | 1.00 | 1.00 | 1.00 | 1.00 |
| Refused/Others/Missing | 1.56(0.83,2.92) | 2.58*(1.13,5.88) | 0.76(0.36,1.57) | 2.07(0.74,5.81) |
| **Mother's schooling** |  |  |  |  |
| No schooling | 1.36*(1.15,1.62) | 1.60*(1.15,2.23) | 1.01(0.80,1.25) | 0.91(0.66,1.27) |
| Primary | 1.20(0.99,1.45) | 1.13(0.79,1.62) | 1.11(0.86,1.42) | 1.22(0.87,1.72) |
| Secondary or Higher (reference) | 1.00 | 1.00 | 1.00 | 1.00 |
| **Caste** |  |  |  |  |
| Scheduled Caste | 1.05(0.86,1.28) | 0.88(0.63,1.23) | 1.10(0.83,1.45) | 1.05(0.68,1.64) |
| Scheduled Tribe | 1.12(0.90,1.39) | 0.99(0.70,1.41) | 1.31(0.98,1.75) | 1.08(0.65,1.79) |
| Other Backward Class | 1.09(0.92,1.30) | 0.89(0.68,1.17) | 1.21(0.95,1.53) | 1.18(0.81,1.71) |
| Others (reference) | 1.00 | 1.00 | 1.00 | 1.00 |
| **Religion** |  |  |  |  |
| Hindu | 0.98(0.71,1.34) | 0.67(0.41,1.08) | 1.43(0.99,2.07) | 2.72*(1.40,5.30) |
| Muslim | 1.02(0.72,1.44) | 0.72(0.42,1.24) | 1.37(0.91,2.07) | 1.78(0.86,3.68) |
| Others (reference) | 1.00 | 1.00 | 1.00 | 1.00 |
| **Wealth quintiles** |  |  |  |  |
| Poorest | 2.21*(1.62,3.01) | 2.19*(1.33,3.58) | 1.77*(1.12,2.79) | 1.08(0.56,2.06) |
| Poorer | 2.14*(1.60,2.86) | 2.16*(1.36,3.43) | 1.75*(1.13,2.70) | 1.27(0.69,2.35) |
| Middle | 1.95*(1.45,2.62) | 1.95*(1.23,3.10) | 1.58*(1.02,2.46) | 1.20(0.65,2.20) |
| Richer | 1.64*(1.24,2.18) | 1.63*(1.05,2.55) | 1.53(0.99,2.33) | 1.20(0.65,2.24) |
| Richest (reference) | 1.00 | 1.00 | 1.00 | 1.00 |
| **Urban-rural residence** |  |  |  |  |
| Urban (reference) | 1.00 | 1.00 | 1.00 | 1.00 |
| Rural | 0.99(0.82,1.19) | 1.11(0.80,1.54) | 0.96(0.77,1.21) | 0.85(0.61,1.19) |
| **State-region** |  |  |  |  |
| North | 1.45*(1.10,1.92) | 1.13(0.75,1.70) | 2.02*(1.33,3.07) | 1.03(0.55,1.94) |
| Centre | 1.88*(1.45,2.45) | 1.18(0.80,1.72) | 2.75*(1.84,4.11) | 1.19(0.66,2.13) |
| East | 1.01(0.75,1.34) | 0.69(0.45,1.06) | 1.60*(1.03,2.49) | 0.83(0.43,1.57) |
| Northeast | 1.67*(1.21,2.29) | 0.99(0.62,1.59) | 3.26*(1.99,5.31) | 2.21*(1.07,4.60) |
| West | 0.99(0.69,1.44) | 0.91(0.52,1.60) | 1.30(0.77,2.18) | 0.82(0.36,1.91) |
| South (reference) | 1.00 | 1.00 | 1.00 | 1.00 |
| ***Note****: OR: odds ratio; * p < 0.05; CI: Confidence interval* | | | | |
