# Supplementary material for: Effect of preterm birth on early neonatal, late neonatal, and postneonatal mortality in India
Source: PLOS Glob Public Health. 2022 Jun 28;2(6):e0000205. doi: 10.1371/journal.pgph.0000205 (PMC10021707; doi:10.1371/journal.pgph.0000205)
Supplement: S7 Table — Note: OR: odds ratio; * p < 0.05; CI: Confidence interval. (DOC) [file pgph.0000205.s008.doc]

| **S7 Table. Mother fixed-effects multivariable binary logistic regression results for neonatal deaths (NND) and infant deaths (IND) for all births in last five years, NFHS-4, India, 2015-16.** | | |
| --- | --- | --- |
| **Variable & category** | **NND**  **(n=8565)** | **IND**  **(n=7333)** |
|  | **Odds ratio (95%CI)** | **Odds ratio (95%CI)** |
| **Index birth preterm** |  |  |
| No (reference) | 1.00 | 1.00 |
| Yes | 7.99*(6.34,10.07) | 6.77*(5.24,8.75) |
| **Birth order (BO) and birth interval (BI)** |  |  |
| First birth order | 5.72*(4.32,7.59) | 4.56*(3.35,6.19) |
| BO 2 or 3 and BI <24 months | 1.63*(1.26,2.10) | 1.56*(1.18,2.06) |
| BO 2 or 3 and BI 2 or 3 and ≥24 months | 2.38*(1.85,3.05) | 2.15*(1.63,2.84) |
| BO ≥4 and BI <24 months | 0.93(0.74,1.17) | 0.93(0.74,1.18) |
| BO ≥4 and BI ≥24 months (reference) | 1.00 | 1.00 |
| **Index child c-section** |  |  |
| No (reference) | 1.00 | 1.00 |
| Yes | 0.47*(0.36,0.61) | 0.43*(0.32,0.58) |
| **Index birth wanted** |  |  |
| No | 2.89*(2.32,3.61) | 3.06*(2.38,3.93) |
| Yes (reference) | 1.00 | 1.00 |
| **Sex of child** |  |  |
| Male (reference) | 1.00 | 1.00 |
| Female | 0.59*(0.53,0.65) | 0.73*(0.65,0.81) |
| **Mother's age at conception** |  |  |
| <20 years | 1.22(0.97,1.54) | 1.09(0.85,1.40) |
| 20-24 years (reference) | 1.00 | 1.00 |
| 25-29 years | 0.93(0.77,1.13) | 0.87(0.69,1.09) |
| ≥30 years | 0.75(0.51,1.09) | 0.55*(0.34,0.87) |
| ***Note****: OR: odds ratio; * p < 0.05; CI: Confidence interval* | | |
