# Supplementary material for: Effect of preterm birth on early neonatal, late neonatal, and postneonatal mortality in India
Source: PLOS Glob Public Health. 2022 Jun 28;2(6):e0000205. doi: 10.1371/journal.pgph.0000205 (PMC10021707; doi:10.1371/journal.pgph.0000205)
Supplement: Attachment — Submitted filename: Reply.docx [file pgph.0000205.s009.docx]

Editorial Board,

PLOS Global Public Health

***Sub: A point-by-point response to editor/reviewer’s comments***

Dear Drs. Kyobutungi and Pai, and fellow Editorial Board members,

Thank you so much for considering our paper entitled **Effect of preterm birth on early neonatal, late neonatal, and postneonatal mortality in India**" for possible publication in the PLOS Global Public Health and for forwarding the thoughtful comments of the reviewers to us. We have made full use of editor/reviewer comments while revising our paper. Please find below a point-by-point response to the editor/reviewer’s comments. We continue to feel that this research makes a very important contribution to knowledge on the relationship between preterm birth and neonatal death in India, and hope that you find the revised paper suitable for publication in PLOS Global Public Health.

Yours Sincerely,

Authors

**Editor’s Comments**

1.Please amend your detailed Financial Disclosure statement. This is published with the article, therefore should be completed in full sentences and contain the exact wording you wish to be published.

i) Please include all sources of funding (financial or material support) for your study. List the grants (with grant number) or organizations (with url) that supported your study, including funding received from your institution.

**Reply:** We have included all sources of funding, and rephrased this statement.

ii). State the initials, alongside each funding source, of each author to receive each grant.

**Reply:** The initials of the funding recipient (AR) have been added to the financial disclosure statement.

iii). State what role the funders took in the study. If the funders had no role in your study, please state: “The funders had no role in study design, data collection and analysis, decision to publish, or preparation of the manuscript.”

**Reply:** The funders had no role in study design, data collection and analysis, decision to publish, or preparation of the manuscript, and this has been noted accordingly in the financial disclosure statement.

iv). If any authors received a salary from any of your funders, please state which authors and which funders.

**Reply:** None of the authors received salary from the funder.

2. Please send a completed 'Competing Interests' statement, including any COIs declared by your co-authors. If you have no competing interests to declare, please state "The authors have declared that no competing interests exist". Otherwise please declare all competing interests beginning with the statement "I have read the journal's policy and the authors of this manuscript have the following competing interests:"

**Reply:** The authors have declared that no competing interests exist.

3. Please note that your Data Availability Statement is currently missing a direct link to access each database. If your manuscript is accepted for publication, you will be asked to provide these details on a very short timeline. We therefore suggest that you provide this information now, though we will not hold up the peer review process if you are unable.

**Reply:** We have provided a direct link to access each database.

4. We do not publish any copyright or trademark symbols that usually accompany proprietary names, eg (R), (C), or TM  (e.g. next to drug or reagent names). Therefore please remove all instances of trademark/copyright symbols throughout the text, including No ®, Birth orders ≥4 order and interval ≥24 months®, Yes ®, Male ®, 20-24 years ® on pages 14 and 15.

**Reply:** We have removed all instances of trademark/copyright symbols throughout the text.

5. We have noticed that you have uploaded supporting information but you have not included a list of legends. Please add a full list of legends for all supporting information files (including figures, table and data files) after the references list.

**Reply:** We have added a full list of legends for all the supporting information files.

**Reviewer’s comments**

**Reviewer #1**: Kannaujiya et al., have presented an interesting study on the association of preterm birth with early, late and post neonatal death in India. The study relies on the data from NFHS-4 survey and reports that children born preterm were four times more likely to die in the early or late neonatal periods. The authors have also listed the limitations of the study, and this study addresses some key questions. However, it will be great if the authors can have more clarity for the following points:

1. Preterm birth can be caused due to multiple factors and can have long-term effect. Here the authors need to clarify if the neonates were (a) small for gestational age, (b) IUGR, (c) extreme, moderate or late preterm? The authors have specified that the NFHS-4 has time reported in months and not weeks, but even from that information the extreme or moderate preterm can be estimated.

**Reply**: Thank you so much for your comment. As suggested we have also estimated the effect of early preterm (i.e, duration of pregnancy <=7 months) and late preterm birth (i.e, duration of pregnancy=8 months) on each outcome variables. The results are shown in Figure 4.

2. Were the preterm spontaneous, or due to preeclampsia or PPROM? This classification is not addressed in the manuscript.

**Reply**: Thank you so much for your comment. Such classification is not available in the data, and we have now noted this in the limitations section.

3. The association with hospitalization and neonatal care is also not accounted for in the manuscript. It will also be good to report the neonatal death in urban versus rural settings and the neonatal care available.

**Reply:** Thank you so much for your comment. As suggested we have analyzed effect of preterm births on childhood mortality stratified by urban-rural residence. In this analysis, preterm births were significantly associated with higher odds of childhood mortality in both rural and urban area; these new findings are now presented in Figure 5. Unfortunately, the data used in this study are a population-based social and demographic health survey, and do not include to neonatal hospitalization or neonatal care for all births in last five years. T

4. Are only singleton pregnancies considered in this study?

**Reply**: Yes, we have included only singleton births for the analysis, as noted in Figure 2.

5. Why was the maternal age considered from 15-49 years and not the traditionally used threshold of 18 years? The chances of preterm is higher for women <18 years of age.

**Reply**: The study included women of reproductive age. In a country like India, where first births quickly follow marriage, and where child marriage is an ongoing concern for policy makers (and has a prevalence of 27% in these data), excluding women below age 18 from the analysis will not give the complete picture.

6. The IRB numbers are not specified in the manuscript. It will be good to include them in the methods section.

**Reply**: This manuscript is a secondary analysis of publicly available, deidentified data from the Indian Demographic Health Survey. The ethical review board of the University of California San Diego approved ethical exemption, and we have included this number in our ethics statement.

**Reviewer #2**: This is the first study on analysis of association of preterm birth and ENND, LNND, and PNND with the findings are very useful for India, especially for the LICs and LMICs. The huge sample size of over 0.2 million births that occurred in 5 years preceding one of the largest population-based representative household surveys is very convincing as seen in the findings of study. Preterm births were also associated with higher risk of dying in the early neonatal, late neonatal, and post-neonatal periods in the family fixed-effects regressions.

Children who were born preterm were four times more likely to die in the early neonatal or late neonatal periods, and more than 1.7 times more likely to die in the post-neonatal period. There are many significant mother related factors as control variables in data analysis to see how important these variables are in contribution to the ENND, LNND, and PNND. ENND among preterm births as compared with no preterm births. However, some comments that authors should take into considerations for possible revision or a further writing:

- Study design was not mentioned (cross sectional or case control study design?) and I do suggest that author should clarify it in the Abstract and Materials and Methods section. Defined study design will help reader know how they could understand about the aim of study as well as the methods for statistical analysis of data.

**Reply**: Thank you so much for your suggestion. We have included study design at appropriate places in the revised text, including the Abstract and Materials and Methods.

- My question is why the child related factors and their death causes of children (before and after neonatal period) were not included as control variables as well as modified risk factors in the study. Authors should add them in this manuscript (if any) and put these data in your additional analysis, because child related factors are also important to see other child biological factors leading these child deaths together with their mother related factors. Otherwise, author should say this is limitation of study that these data were not available.

**Reply:** Thank you for your suggestion. Our study includes a number of child related variables, such as birth order and birth interval, cesarean section, wantedness of pregnancy, and sex of child. As the information on antenatal care (ANC) and its components were only available for most recent births, we could not include ANC related variables in the main models. However, we included the ANC related variables, such as four or more ANC (no, yes), >=2 tetanus injection (no, yes) and >=100 iron folic acid tablets (no, yes), in the third sensitivity analysis in which we restricted the regressions among the most recent births. We could not include birth weight (an important child health variable) because information on birth weight were not available for a large proportion of births (25%) – this is now noted in the manuscript text. We also could not include the causes of deaths in the study because no information was collected on causes of deaths in NFHS-4. However, as suggested, we have now listed these in the limitation of our study.

- The findings from Table 2 (Mother fixed-effects multivariable logistic regression results for ENND, LNND, and PNND for all births in last five years, NFHS-4, India, 2015-16) are very important as risk factors of preterm birth (Birth order, child C-section, sex of child....) and they should be shortly presented in the abstract, not only showing single association between neonatal mortality and preterm birth.

**Reply**: Thank you so much for your suggestion. We have included interpretation of mother fixed-effects in the abstract.

- The author calculated OR (see Table 2) to see risk factors of neonatal mortality as preterm birth or mother related factors etc. Is this study design therefore defined as case control for data analysis? Please explain to clarify.

**Reply**: The NFHS-4 followed a cross-sectional study design. Odds ratios can also be estimated in cross-sectional data using logistic regressions.

- Do authors have any other evidences to show Ethical approval, for this study, by the Institutional review Boards of both IIPS and ICF? Looking at the website: <https://dhsprogram.com/> seems not enough information about ethical consideration.

**Reply**: The NFHS-4 final report, available here (<https://dhsprogram.com/pubs/pdf/FR339/FR339.pdf>), includes the following statement “*The protocol for the NFHS-4 survey, including the content of all the survey questionnaires, was approved by the IIPS Institutional Review Board and the ICF Institutional Review Board. The protocol was also reviewed by the U.S. Centers for Disease Control and Prevention (CDC).”* We have now included this citation in the Ethics section of the manuscript.
