# Supplementary material for: Effect of preterm birth on early neonatal, late neonatal, and postneonatal mortality in India
Source: PLOS Glob Public Health. 2022 Jun 28;2(6):e0000205. doi: 10.1371/journal.pgph.0000205 (PMC10021707; doi:10.1371/journal.pgph.0000205)
Supplement: Attachment — Submitted filename: Response to Reviewers Final.docx [file pgph.0000205.s010.docx]

Dated: April 5, 2022

To

Dr. Zohra S. Lassi

Academic Editor

PLOS Global Public Health

***Sub: A point-by-point response to editor/reviewer’s comments***

Dear Dr. Lassi

Thank you so much for allowing us to do the second revision of our paper titled “**Effect of preterm birth on early neonatal, late neonatal, and postneonatal mortality in India**” for possible publication in the PLOS Global Public Health. We have made the necessary changes in the paper, and hope that you find the revised version suitable for publication in PLOS Global Health.

Yours Sincerely

Authors

**Journal Requirements:**

2. Your co-authors, Ashish Kumar Upadhyay ([ashu100789@gmail.com](mailto:ashu100789@gmail.com)), Anita Raj ([anitaraj@ucsd.edu](mailto:anitaraj@ucsd.edu)), and K S James ([ksjames@iipsindia.ac.in](mailto:ksjames@iipsindia.ac.in)), have not confirmed authorship of the manuscript. We have resent them the authorship confirmation email; however please check that the above email address for them is correct and follow up personally to ensure they confirm. Please note that we cannot pass your manuscript to Production until we have received confirmations from all co-authors.

**Response**: *Thank you for reminding us to confirm the authorship. All three authors have confirmed their authorship of the manuscript.*

2. Please review your reference list to ensure that it is complete and correct. If you have cited papers that have been retracted, please include the rationale for doing so in the manuscript text, or remove these references and replace them with relevant current references. Any changes to the reference list should be mentioned in the rebuttal letter that accompanies your revised manuscript. If you need to cite a retracted article, indicate the article’s retracted status in the References list and also include a citation and full reference for the retraction notice.

**Response**: *Thank you for reminding us to review the reference list. We have cross validated the citations in the text and their corresponding references. We have replaced the citation no. 30 with current reference.*

**Review Comments to the Author**

**Reviewer #1**: The authors have done a great work in revising the manuscript and addressing all the comments. I have minor comments related to the work:

1. In Table 2, what does the term 'reference' suggest? It's repeated a number of times in the table, but is not indicated in the legends.

**Response**: *In table 2, reference indicates the category with which other categories of a variable are compared i.e. reference category. We have stated it clearly in the revised text.*

2. In the multivariable logistic regression model, do the authors adjust for mother's age at the time of birth?

**Response**: *Yes, we have adjusted mother’s age at conception instead of mother’s age at birth. We have correctly stated this point in the revised text.*

**NOTE**

While revising your submission, please upload your figure files to the Pre-flight Analysis and Conversion Engine (PACE) digital diagnostic tool, https://pacev2.apexcovantage.com/. PACE helps ensure that figures meet PLOS requirements. To use PACE, you must first register as a user. Registration is free. Then, login and navigate to the UPLOAD tab, where you will find detailed instructions on how to use the tool. If you encounter any issues or have any questions when using PACE, please email PLOS at figures@plos.org. Please note that Supporting Information files do not need this step.

**Response**: *We have uploaded the figure using the PACE digital diagnostic tool.*
